# Unrecognized circulation of SAT 1 foot-and-mouth disease virus in cattle herds around Queen Elizabeth National Park in Uganda

**DOI:** 10.1186/s12917-015-0616-1

**Published:** 2016-01-06

**Authors:** Moses Tefula Dhikusooka, Chrisostom Ayebazibwe, Alice Namatovu, Graham J. Belsham, Hans Redlef Siegismund, Sabenzia Nabalayo Wekesa, Sheila Nina Balinda, Vincent B. Muwanika, Kirsten Tjørnehøj

**Affiliations:** National Animal Disease Diagnostics and Epidemiology Centre, Ministry of Agriculture Animal Industry and Fisheries, P. O. Box 513, Entebbe, Uganda; Department of Biotechnical and Diagnostic Sciences, College of Veterinary Medicine, Animal Resources and Biosecurity, Makerere University, P. O. Box 7062, Kampala, Uganda; National Veterinary Institute, Technical University of Denmark, Lindholm, Kalvehave, DK 4771 Denmark; Department of Biology, University of Copenhagen, Ole Maaløes Vej 5, DK 2200 Copenhagen N, Denmark; Foot-and-Mouth Disease Laboratory, Ministry of Livestock Development, P. O. Box 18021, Embakasi, Nairobi Kenya; Department of Environmental Management, College of Agricultural and Environmental Sciences, Makerere University, P. O. Box 7062, Kampala, Uganda

**Keywords:** Livestock-wildlife interface, Foot-and-mouth disease virus, SAT 1, Young cattle, Uganda

## Abstract

**Background:**

Foot-and-mouth disease (FMD) is endemic in Uganda in spite of the control measures used. Various aspects of the maintenance and circulation of FMD viruses (FMDV) in Uganda are not well understood; these include the role of the African buffalo (*Syncerus caffer*) as a reservoir for FMDV. To better understand the epidemiology of FMD at the livestock-wildlife-interface, samples were collected from young, unvaccinated cattle from 24 pastoral herds that closely interact with wildlife around Queen Elizabeth National Park in Uganda, and analysed for evidence of FMDV infection.

**Results:**

In total, 37 (15 %) of 247 serum samples had detectable antibodies against FMDV non-structural proteins (NSPs) using a pan-serotypic assay. Within these 37 sera, antibody titres ≥ 80 against the structural proteins of serotypes O, SAT 1, SAT 2 and SAT 3 were detected by ELISA in 5, 7, 4 and 3 samples, respectively, while neutralizing antibodies were only detected against serotype O in 3 samples. Two FMDV isolates, with identical VP1 coding sequences, were obtained from probang samples from clinically healthy calves from the same herd and are serotype SAT 1 (topotype IV (EA-I)). Based on the VP1 coding sequences, these viruses are distinct from previous cattle and buffalo SAT 1 FMDV isolates obtained from the same area (19–30 % nucleotide difference) and from the vaccine strain (TAN/155/71) used within Uganda (26 % nucleotide difference). Eight herds had only one or a few animals with antibodies against FMDV NSPs while six herds had more substantial evidence of prior infection with FMDV. There was no evidence for exposure to FMDV in the other ten herds.

**Conclusions:**

The two identical SAT 1 FMDV VP1 sequences are distinct from former buffalo and cattle isolates from the same area, thus, transmission between buffalo and cattle was not demonstrated. These new SAT 1 FMDV isolates differed significantly from the vaccine strain used to control Ugandan FMD outbreaks, indicating a need for vaccine matching studies. Only six herds had clear serological evidence for exposure to O and SAT 1 FMDV. Scattered presence of antibodies against FMDV in other herds may be due to the occasional introduction of animals to the area or maternal antibodies from past infection and/or vaccination.

The evidence for asymptomatic FMDV infection has implications for disease control strategies in the area since this obstructs early disease detection that is based on clinical signs in FMDV infected animals.

**Electronic supplementary material:**

The online version of this article (doi:10.1186/s12917-015-0616-1) contains supplementary material, which is available to authorized users.

## Background

Foot-and-mouth disease (FMD) is a highly contagious viral disease affecting a wide range of domestic and wild cloven-hoofed animals [1, 2]. It is known to cause substantial economic losses, directly from the effect of the virus on animal health and indirectly through control efforts including quarantines and trade restrictions [2].

The disease is caused by infection with a single stranded, positive sense, RNA virus (FMDV) belonging to the genus *Aphthovirus* within the family *Picornaviridae*. This virus occurs in seven distinct serotypes namely: O, A, C, Asia 1, SAT 1, SAT 2 and SAT 3, each having multiple strains [3, 4]. All serotypes, except Asia 1, have been reported in eastern Africa, however, serotype C was last detected in Kenya in 2004 and may be extinct [5, 6]. Serotypes O and A are considered endemic in most parts of Africa, while the three SAT serotypes have usually been restricted to sub-Saharan Africa [7], where they are commonly diagnosed [7–9]. However, incursions of SAT 1 into the Middle East (1961–1965 & 1970), and SAT 2 into Saudi Arabia (2000), Libya (2003) and more recently into Egypt and Libya (2012) have been reported [7, 10].

The maintenance of the three FMDV SAT serotypes in cattle in southern and eastern Africa has been associated with contact with wildlife, especially African buffalo (*Syncerus caffer*) [11]. FMDV has been demonstrated to persist in individual buffalo for up to 4–5 years and in isolated buffalo herds for up to 24 years [11]. In southern Africa, it has, in some cases, been concluded that buffalo have been the source of FMDV infections in cattle and impala (*Aepyceros melampus*) [8, 12, 13]. As a result, FMD control policies in southern Africa are centred on isolating the buffalo, in the National Parks and game reserves, away from the surrounding livestock populations, and vaccinating cattle kept in buffer zones around these areas against the FMDV serotypes that are carried by the nearby buffalo [14].

In eastern Africa, FMD was first reported in cattle in 1932 [15] and since then, the marketability of livestock and animal products within, and outside, the region has been negatively affected [16]. The maintenance of FMDV in the region has been attributed to various factors including: the presence of numerous wildlife reservoirs [7, 17–19], communal and pastoral grazing systems that enable contact between livestock and wildlife [20], poor diagnostic capacity in the region [9], emergence of new strains [21–23], transboundary animal movements and traditional cultural practices such as the exchange of live animals for dowry and gifts [24, 25]. East African countries have tried to control the disease mainly by quarantine and post outbreak vaccination. However, these measures have been difficult to enforce and suboptimal use of vaccines [26] has resulted in large parts of the FMDV susceptible animal populations remaining at risk [9, 27]. Recently, the countries in this region have established a network to combine their control efforts to move towards freedom from FMD by 2020 following the progressive control pathway (PCP) for FMD as described [28]. The PCP defines six steps in the process of achieving freedom from FMD including effective monitoring of circulating serotypes, vaccination and improved biosecurity measures [28]; this strategy has been adopted by all countries in the region, including Uganda [29].

In Uganda, SAT 1 FMDV was first reported in 1959, and since then, FMD outbreaks caused by serotypes O, A, SAT 1 and SAT 2 [7, 9, 24, 27, 30–33] have been regularly reported in livestock. In addition, for the first time, serotype SAT 3 FMDV has been recently isolated from a clinically healthy long horned Ankole calf shortly after its introduction into the area close to the Queen Elizabeth National Park (QENP) [34]. Ugandan buffalo are only infrequently sampled for FMDV, and thus the virus has only been isolated from them during two studies in QENP; these yielded serotypes SAT 1 and SAT 3 in 1997 [35] and SAT 1 and SAT 2 in 2007 [36]. Moreover, in a post-outbreak study conducted on adult cattle and goats in the same area, concurrently with the 2007 buffalo sampling, only serotype O FMDV was isolated. Furthermore, 11 herds of cattle had animals with higher antibody titres against serotype O than against the SAT serotypes, while only one had convincing serological evidence of infection with SAT FMDV [22, 31]. Thus, although wildlife within the Ugandan National Parks are often blamed for being the source of FMD outbreaks in their surroundings [37], this could not be proven for the major 2006 FMD outbreak in the QENP area [31, 36]. Moreover, although it is clear that FMDV can pass from buffalo to cattle [13], recent attempts in Uganda and Kenya to establish the role of the African buffalo in the epidemiology of FMD in domestic species in eastern Africa have so far not settled this [36, 38].

**Fig. 1:**
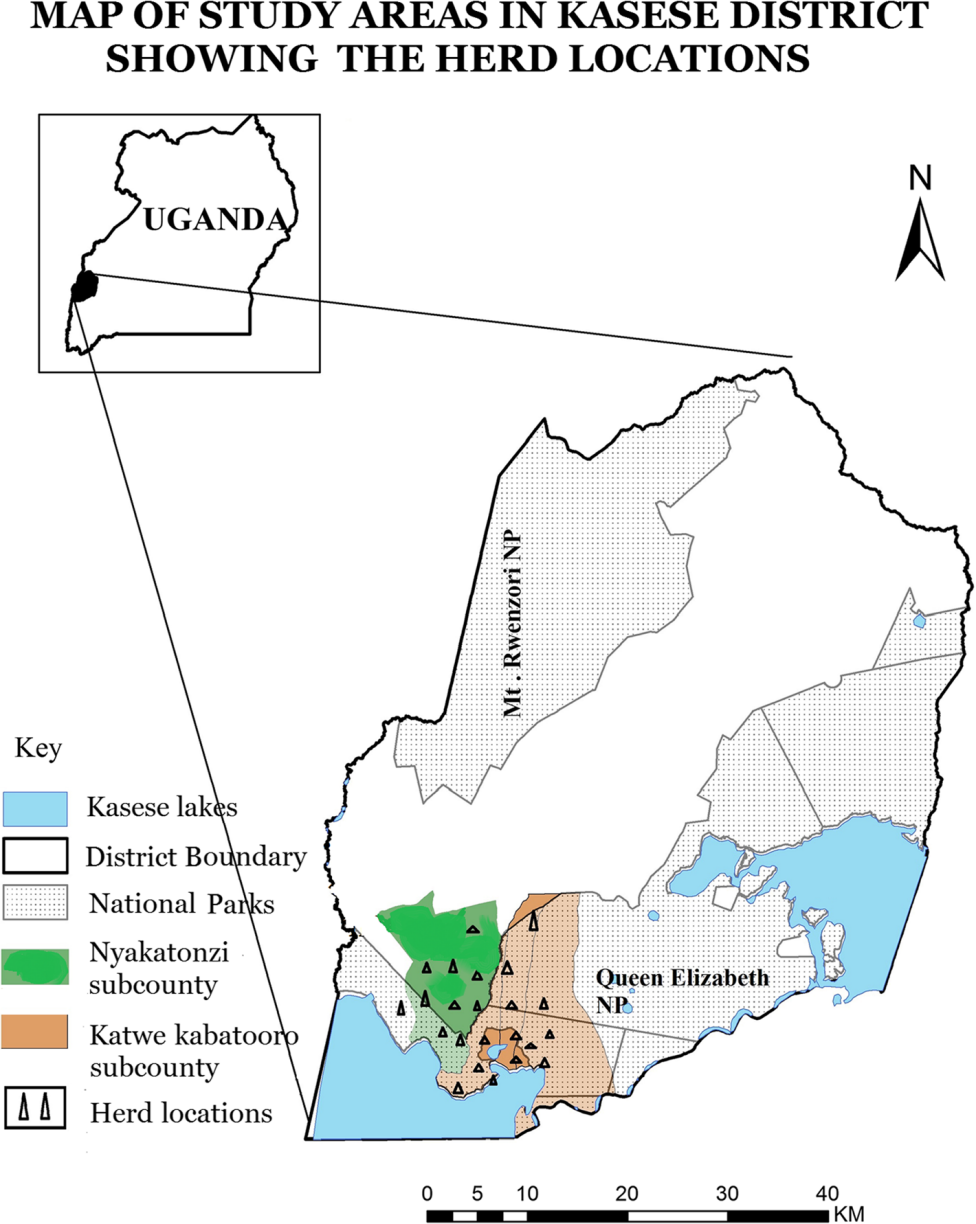
Map of the study area: Queen Elizabeth National Park (QENP) and its surroundings. Generated using QGIS, version 2.6.1

The objective of this study was to assess the presence of FMDV in young, unvaccinated cattle grazing in close proximity to African buffalo in the QENP (Fig. 1) and to establish whether these buffalo play a major role in the maintenance of FMD in livestock around the National Park.

## Methods

This study was designed in compliance with REFLECT guidelines and was approved by the Ugandan Ministry of Agriculture, Animal Industries and Fisheries (MAAIF) (Reference LHE 199/01, Uganda) and Makerere University (MU) “School Higher Degrees and Research Committee”. The sampling was carried out by MAAIFs National Animal Disease Diagnostics and Epidemiology Centre (NADDEC) in collaboration with Kasese District Veterinary Office in accordance to the guidelines from MAAIF. The study included meetings for the herd owners where the purpose of the project was described and informed consent to the sampling, and to participation in the questionnaire survey, was obtained from the individual herd owners.

### Sampling approach

A cross-sectional study was conducted in Katwe Kabatooro and Nyakatonzi subcounties of Kasese district in Uganda (Fig. 1) during August-September 2011 to assess the presence of FMDV and antibodies against this virus. The sampled herds were usually grazed communally close to (and inside) the QENP, where the animals, according to the herd owners, regularly moved/grazed/drank within 2–10 m of wildlife. FMD was last reported from this area in 2006–2007, and no vaccinations against FMDV had been carried out in this area since 2007.

The two sampled subcounties had an estimated cattle population of 30,000 head of cattle distributed in approximately 50 herds (personal communication, DVO Dr. V. Kalule, based on [39]). Moreover, it was estimated that approximately 1/6 of these, i.e. 5,000, were unvaccinated 6-24-month-old cattle. A sample size of 296 was calculated using Win Episcope 2.0 software [40] at a 5 % precision level and 95 % confidence level and assuming antibody prevalence of approximately 12 % [41].

At the sampling, both unstabilised blood and oropharyngeal fluid (OP) samples were collected from each of 247 heads of 6-24-month-old cattle. The cattle belonged to 24 settled pastoralists, who in total owned 1171 cattle within this age-cohort, and in each herd, sampled animals were randomly selected. Due to the field conditions, the calculated number of samples was not entirely met; however, the number of cattle in the sampled age-group in the 24 sampled herds was also lower than estimated (2400), so this was evaluated as adequate.

The OP samples were immediately divided into two aliquots: one placed in phosphate buffered saline (PBS) and another in RLT buffer (Qiagen, Hilden, Germany) and kept in liquid nitrogen until stored at −80 °C in the laboratory, while serum was extracted in the field and kept on ice until stored at −20 °C in the laboratory.

Information on: vaccination history, laboratory confirmed FMD outbreaks, unreported observation of clinical symptoms of FMD, farming characteristics, history of contact between cattle and wildlife, age group mostly affected in the event of FMD outbreaks and FMD control methods was collected from the herd owners using a semi-structured questionnaire (Additional file 1).

### Serology

Sera were screened for the presence of antibodies against FMDV NSPs using PrioCHECK® FMDV NS ELISA test kits (Prionics, Lelystad, The Netherlands) according to the manufacturer’s instructions. Positive samples were tested (at 1:10 dilution) for antibodies against the seven different FMDV serotypes using solid phase blocking ELISAs (SPBEs) [42]. Results were read as the optical density (OD) of wells with test samples and expressed as a percentage of the mean optical density (ODP) of four wells with a known negative control serum. Sera were considered positive, if the ODP was < 50 % for serotypes O, SAT 1, SAT 2 and SAT 3, < 45 % for serotype A, and < 35 % for serotypes C and Asia 1. Due to expected cross-reactions, all sera giving positive reactions in the initial screening were titrated in two-fold dilution series from 1/10 to 1/1280 and titres expressed as the reciprocal of the highest positive dilution [42]. Nineteen samples with titres ≥ 80 in the SPBEs were tested in the relevant virus neutralization tests (VNTs) as described in the OIE Manual [43], while twelve sera with SPBE titres ≤ 40 were not tested further. FMDV strains used for VNT comprised O Manisa, A Iraq 24/64, C Noville, Asia 1 Shamir, SAT 1 BOT/68, SAT 2 ZIM 5/81 and SAT 3 ZIM 4/81; VNT titres ≥ 45 were considered positive, titres of 15–44 inconclusive and titres ≤ 14 negative.

### Detection of FMDV RNA and isolation of FMDV from OPs

Thirty two OP samples collected from the animals with antibodies against FMDV NSP were initially screened for FMDV RNA using the 5'-untranslated region (UTR) targeted quantitative reverse transcription PCR (RT-qPCRs) [43, 44] and 22 of these with threshold cycle values (C_T_ values) ≤ 39 were inoculated onto primary bovine thyroid cells (BTY) and propagated through two cell culture passages [32, 36, 43]. Cultures with cytopathic effect (CPE) were harvested and stored at −80 °C. The 3D coding region targeted RT-qPCR assay was then performed on RNA extracted from the cell harvests as described previously [45] and samples with C_T_ < 32 were interpreted as FMDV RNA positive while C_T_ values ≥ 32 were considered negative [45, 46]. The presence of FMDV in CPE-positive harvests was analysed using an in-house antigen ELISA, which also determined the serotype of positive isolates as previously described [32, 36, 43].

### Sequencing of VP1 coding region from FMDV isolates

RNA was extracted from FMDV antigen positive cell culture harvests as described above, and cDNA synthesized using Ready-To-Go You-Prime First-strand Beads (GE Healthcare Life Sciences, Uppsala, Sweden) with random hexamer primers (pd(N)_6_) [27]. The cDNA was diluted to 150 μl from which an aliquot was used for VP1 coding region amplification as follows: 5 μl 10X PCR buffer, 4 μl 25 mM MgCl_2_, 25.1 μl dH_2_O, 0.5 μl Amplitaq Gold® DNA Polymerase (Applied Biosystems Foster City, USA), 0.4 μl 10 mM dNTPs, 5.0 μl of each of forward (13 KPN-100 [5´GGGTGGBBGTSTWMCAGRTSACMGACAC 3´]) and reverse (FMD KS2B58 [5´-ACAGCGGCCATGCACGACAG 3´] primers, where B = (C or G or T); S = (C or T); W = (A or T); M = (A or C) and R = (A or G) [47], with 5 μl of cDNA [36, 48].

The PCR was run in a thermocycler (Gene Amp-PCR system 3700 version 3.0 Applied Biosystems) using the touchdown technique as described [48]. The PCR products (731 bp) were purified using QIAquick® gel extraction and purification kit (Qiagen, Hilden, Germany), and quantified using a Nanodrop®1000 spectrophotometer (Fisher Scientific, Waltham, MA, USA). Sequencing of the PCR products was performed using a BigDye® Terminator v 3.1 Cycle Sequencing kit (Applied Biosystems, Foster City, CA, USA), using the same primers as used in the PCRs, according to the manufacturer’s instructions, and ran on an automated DNA sequencer (ABI PRISM® 3730 DNA Analyser (Applied Biosystems, Foster City, CA, USA).

### Sequence assembly and analysis

Nucleotide sequences were assembled and edited using SeqMan Pro™ software (DNAStar Lasergene 10.0, Madison, WI, USA). The resulting consensus sequences were compared to others using the Basic Local Alignment Search Tool (BLAST) in MEGA 5 (www.megasoftware.net [49]). They were then aligned with 19 other published nucleotide sequences obtained from GenBank (Table 1) in MUSCLE incorporated in MEGA software version 6.06, and trimmed to correspond to the 657 nt encompassing the complete VP1 coding region. Phylogenetic trees were estimated using the Neighbor Joining (NJ) method [50], with 1000 bootstrap replicate samplings and used to determine the topotype clusters for the aligned sequences. The amino acid sequences were deduced from the nucleotide sequences using the standard genetic code and the extent of amino acid relatedness in the VP1 coding regions determined using the same program in MEGA 6 [51].

**Table 1:** SAT 1 VP1 coding sequences included in this study

Species	Isolate Identity	GeneBank accession no.	Year of sampling	Country	Topotype
Cattle	UGA/116/13**	KP025678	2013	Uganda	IV (EA-1)
Cattle	UGA/161/13**	KP025679	2013	Uganda	IV (EA-1)
Buffalo	UGA/10/70	KF219681	1970	Uganda	IV (EA-1)
Buffalo	UGA/21/70	KF219682	1970	Uganda	IV (EA-1)
Buffalo	UGA/1/97	AY442012	1997	Uganda	VIII (EA-3)
Unknown	UGA/7/99	AY442011	1999	Uganda	IV (EA-1)
Buffalo	UGA/1/07	HM067706	2007	Uganda	IV (EA-1)
Cattle	UGA/13/74	AY442010	1974	Uganda	VII (EA-2)
Cattle	ETH/19/07	FJ98156	2007	Ethiopia	IX
Cattle	ETH/21/07	FJ798157	2007	Ethiopia	IX
Cattle	KEN/11/91	AY441994	1991	Kenya	I (NWZ)
Cattle	KEN/9/91	AY441995	1998	Kenya	I (NWZ)
Cattle	KEN/28/06	HQ267529	2006	Kenya	III (WZ)
Cattle	KEN/66/80	HQ267520	1980	Kenya	I (NWZ)
Cattle	TAN/2/77	AY442008	1977	Tanzania	I (NWZ)
Cattle	TAN/155/71 (Vaccine)	HQ267519	1971	Tanzania	I (NWZ)
Cattle	TAN/19/96	AY442005	1996	Tanzania	I (NWZ)
Cattle	TAN/60/99	AY442002	1999	Tanzania	III (WZ)
Cattle	NIG/2/79	AF431728	1979	Nigeria	VI
Cattle	KNP/196/91	DQ009716	1991	South Africa	II (SEZ)

**: sequences obtained as part of this study

## Results

Clinical signs of FMD were not observed in any of the sampled animals or herds.

### Serology

In total, 37 of 247 sera collected from 24 herds in Nyakatonzi and Katwe Kabatooro subcounties tested positive for antibodies against the NSPs of FMDV (Table 2) giving an overall antibody prevalence of 15 % with herd antibody prevalences ranging from 0 to 60 % (median 11 %; Inter Quartile Range (IQR) 0–26 %). In the 14 positive herds, the antibody prevalences in the NSP antibody test ranged from 6 to 60 % (median 23 %; IQR 16–30 %).

**Table 2:** Summarized results from herds with animals positive for antibodies against FMDV NSPs

Subcounties	Parishes	Herd no.	NSP ELISA Pos/tested (%)	SPBE titre				VNT titre				Serological conclusion herd-level	Cell culture BTY	Antigen ELISA on BTY harvest	3D RT - qPCR (C_T_) on virus harvests	Herd diagnosis*
				O	SAT 1	SAT 2	SAT 3	O	SAT 1	SAT 2	SAT 3					
Katwe	Rwenjubu	H1	6/23 (26)	-	10	-	-	-	-	-		O				+
Kabatooro				640	320	10	40	67	-		28		CPE	-	-	
				160	-	-	-	24	-	-	-		-			
				10	20	-	-		-							
				-	10	-	-		17							
				-	10	-	-	-								
	Kijarukara	H3	3/12 (25)	10	320	10	40	-	28	-	-	(SAT 1)	-			(+)
				-	-	-	-						-			
				-	-	10	10						CPE	-	-	
	Kiganda	H7	3/5 (60)	-	-	-	-						-			+
				10	-	-	10	40	-		17		-			
				10	10	-	10	34	-		28		-			
	Top hill	H19	4/7 (57)	10	P**	-	10	-			24	O				+
				160	20	10	80	136	-	28	-	(SAT 2)	CPE	-	32	
				-	-	80	10	-	-	40						
				10	20	-	80	24	-	28	-					
	Kyakitare	H18	2/10 (20)	10	80	10	10	-	34	28		(SAT 1)	CPE	SAT 1	16	++
				10	40	40	20	-	-				CPE	SAT 1	19	
	Kiganda	H2	1/7 (14)	-	-	-	-						-			(+)
	Rwenjubu	H5	2/7 (29)	-	10	10	10		17				-			(+)
				-	-	-	-									
Nyakatonzi	Muruti	H12	5/18 (28)	-	-	-										(+)
				-	-	-	-									
				-	-	-	-									
				-	-	-	-									
				-	-	-	-									
	Muruti	H15	3/9 (33)	-	-	-	-						-			(+)
				-	10	-	-	-	17	-			-			
				40	-	-	-	-	-	-			-			
	Muruti	H14	1/5 (20)	160	160	80	20	48	24	-	-	O	CPE	-	32	+
	Kisasa	H23	4/25 (16)	160	80	20	320	24	-	-	17	(O)	CPE	-	32	+
				-	160	160	-	-	-	28		(SAT 2)	CPE	-	-	
				40	40	40	-	40	-	-	-					
				20	20	80	40	40	-	-			CPE	-	-	
	Kisasa	H22	1/6 (17)	10	20	-	10	34	-	-	28		CPE	-	-	(+)
	Kisasa	H21	1/17 (6)	-	-	-	-									(+)
	Bwanika	H11	1/13 (8)	-	-	-	-						-			(+)
No. Positive			37/247 (15)	17	20	13	16	3	0	0	0		na	2		
No. with SPBE-titre ≥ 80				5	6	4	3									

-: Negative

*: (+) only few animals with antibodies against the NSPs

+ Herds with more substantial evidence of exposure to FMDV

++ Confirmed evidence of exposure to FMDV

**: Positive in SPBE 1:10 but not titrated due to insufficient sample

Screening of the 37 anti-NSP antibody positive samples in SPBEs resulted in 17, 4, 2, 0, 20, 13 and 16 samples being scored positive for antibodies against serotypes O, A, C, Asia 1, SAT 1, SAT 2 and SAT 3, respectively, while 12 anti-NSP positive samples were negative in all SPBEs (Table 2; data not shown for the testing of antibodies against serotypes A, C and Asia 1). When titrated, the few sera that were identified as positive for antibodies against serotypes A and C in the initial screening (at 1:10 dilution) had titres ≤ 40 and so were considered negative. In contrast, titres ≥ 80 were found in 5/17, 6/20, 4/13 and 3/16 of the samples titrated for antibodies against serotypes O, SAT 1, SAT 2 and SAT 3, respectively (Table 2). In summary, the positive results were obtained from 11 sera collected from 6 different herds. Six of these sera had high antibody titres against one single serotype (O: 1 sample; SAT 1: 2 samples; SAT 2: 2 samples, SAT 3: 1 sample), whereas five sera had high antibody titres against more than one of the serotypes O, SAT 1, SAT 2 and SAT 3 (Table 2).

Neutralising antibodies (as determined by VNT) were only detected against serotype O and only in three animals, while eight, six, five and six sera had inconclusive neutralizing antibody titres against O, SAT 1, SAT 2 or SAT 3, respectively, and all the other tested sera were negative (Table 2). The three VNT positive animals had SPBE antibody titres ≥ 160 and came from three different herds (H1, H14 and H19).

### Diagnostic RT-qPCRs, virus isolation and antigen ELISA

In the 5´UTR RT-qPCR assay, 22 out of the 32 tested OP samples generated C_T_ values ranging from 22 to 39, of which only three samples had C_T_ <32 (22, 23 and 25). Ten of these 22 OP samples produced CPE when inoculated onto primary BTYcells but only two cell culture harvests (UGA/116/13 and UGA/161/13) were found positive in the 3D RT-qPCR assay (defined as positive: C_T_ < 32) and these had C_T_ values of 16 and 19 (indicative of high levels of FMDV RNA). Moreover, only the same two virus harvests were found positive for FMDV antigen in FMDV antigen ELISAs and were identified as serotype SAT 1 (Table 2). These two virus harvests were generated from the OP samples with the lowest C_T_ values in the 5´UTR RT-qPCR assay, and were both collected from herd H18. RNA extracted from cell harvests derived from one OP from each of herds H14, H19 and H23 each had a C_T_ value 32 in the 3D RT-qPCR, which is on the borderline of confidence in detection [46], but no FMDV could be isolated.

### Herd diagnosis

There was no evidence of exposure to FMDV in ten of the 24 herds in this study (H4, H6, H8, H9, H10, H13, H16, H17, H20 and H24) (data not shown), while four herds had antibodies against FMDV NSPs only, either in one animal (H2, H11 and H21) or in five animals (H12) (Table 2). Another four herds had anti-NSP antibody seroprevalences of 17–33 % (1–3 animals) with antibodies against one or more serotypes in 1–2 of these animals (H3, H5, H15 and H22). Six herds had more substantial evidence for exposure to FMDV. This was in the form of higher seroprevalences (43–60 %) of antibodies against FMDV NSPs (H7 and H19), SPBE antibody titres ≥ 80 in more than one animal (H1, H19 and H23), neutralising antibodies in one animal (H1, H14 and H19) and isolation of FMDV from OPs (H18).

### Characterisation of FMDV isolates

**Fig. 2:**
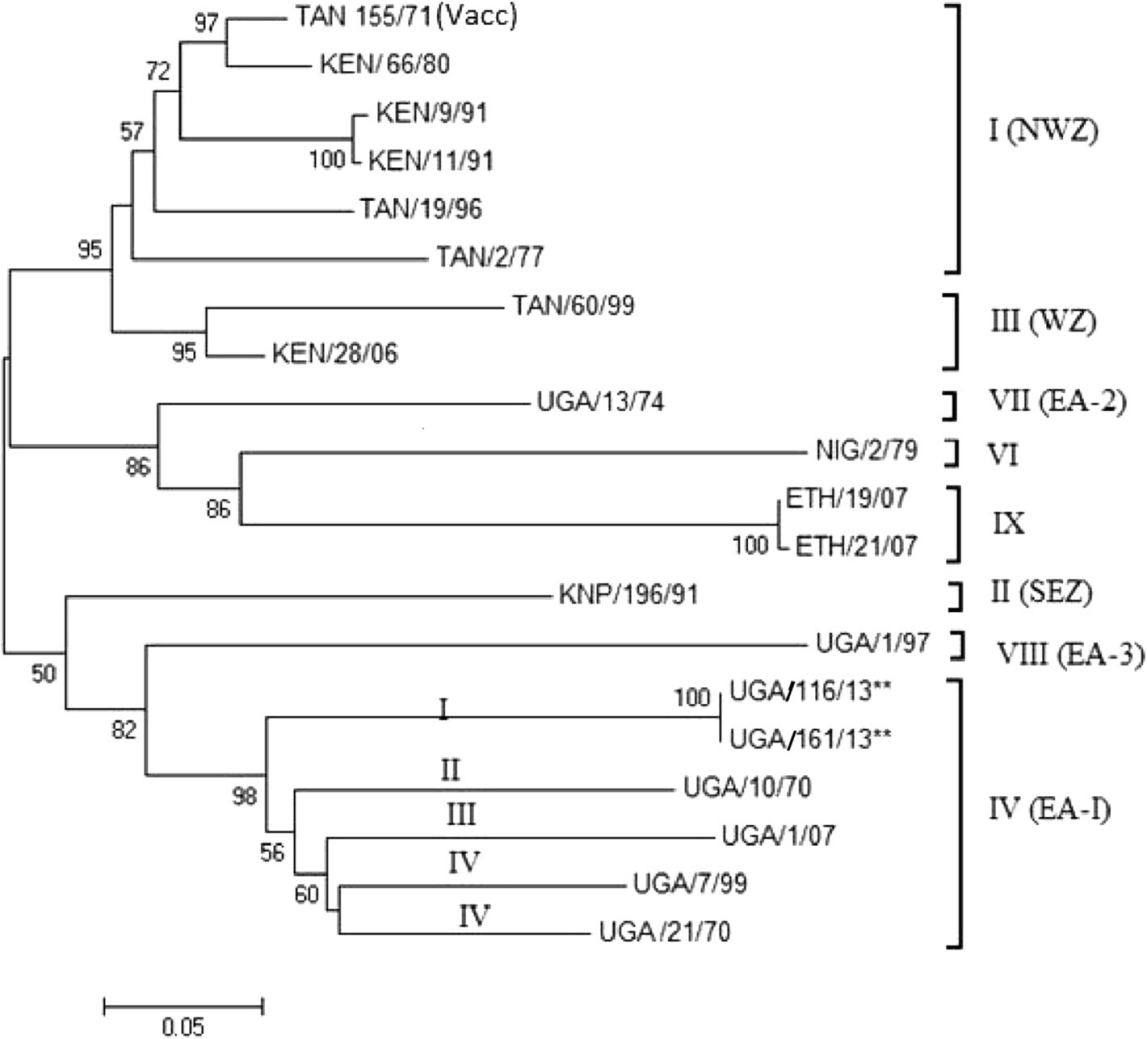
A Neighbor-joining tree showing the relationships between the VP1 coding sequences (657 nt) of the two Ugandan SAT 1 isolates from this study (UGA/116/13 [KPO25678] and 161/13 [KPO 25679] (marked **)) and SAT 1 FMDV VP1 coding sequences from Uganda (UGA), Kenya (KEN), Tanzania (TAN), Ethiopia (ETH), Nigeria (NIG) and Kruger National Park (KNP) in South Africa as obtained from GenBank (http://www.ncbi.nlm.nih.gov/genbank/). Topotypes are indicated according to Tekleghiorghis et al. (2014) [49]. Bootstrap values are indicated. Scale bar indicates nucleotide substitutions per site

Sequencing revealed that the two 657 nucleotide (nt) long regions, from the 731 bp amplicons, corresponding to the VP1 coding sequences of UGA/116/13 and UGA/161/13 were identical. Alignment of these complete VP1 sequences with 18 other African FMDV isolates (Table 1) showed that they belonged to the SAT 1 topotype IV (EA-1) (see Tekleghiorghis et al. [52]), clustered together with three previous Ugandan buffalo isolates of 1970 and 2007 (UGA10/70, UGA/21/70 and UGA/1/07) and also the UGA/7/99 isolate whose species of origin was not identified (Fig. 2). The VP1 sequences of the 2013 isolates had 21 %, 20 % and 24 % nucleotide (nt) difference from these earlier buffalo isolate VP1 sequences, respectively, while their relationship with another Ugandan buffalo isolate from 1997 (UGA/1/97) and an earlier Ugandan SAT 1 cattle isolate (UGA/13/74) was even more distant (nt sequence divergences of 30 % and 31 %, respectively). However, except for the UGA/13/74 strain [topotype VII (EA-2)] and UGA/1/97 [topotype VIII (EA-3)], the various Ugandan isolates represent independent lineages within topotype IV (EA-1) (Fig. 2). The SAT 1 vaccine strain (TAN/155/71) used in Uganda belongs to a different topotype I (NWZ) and differs from the SAT 1 VP1 sequences obtained from this study by 26 % and 21 % at the nucleotide and amino acid level, respectively.

**Fig. 3:**
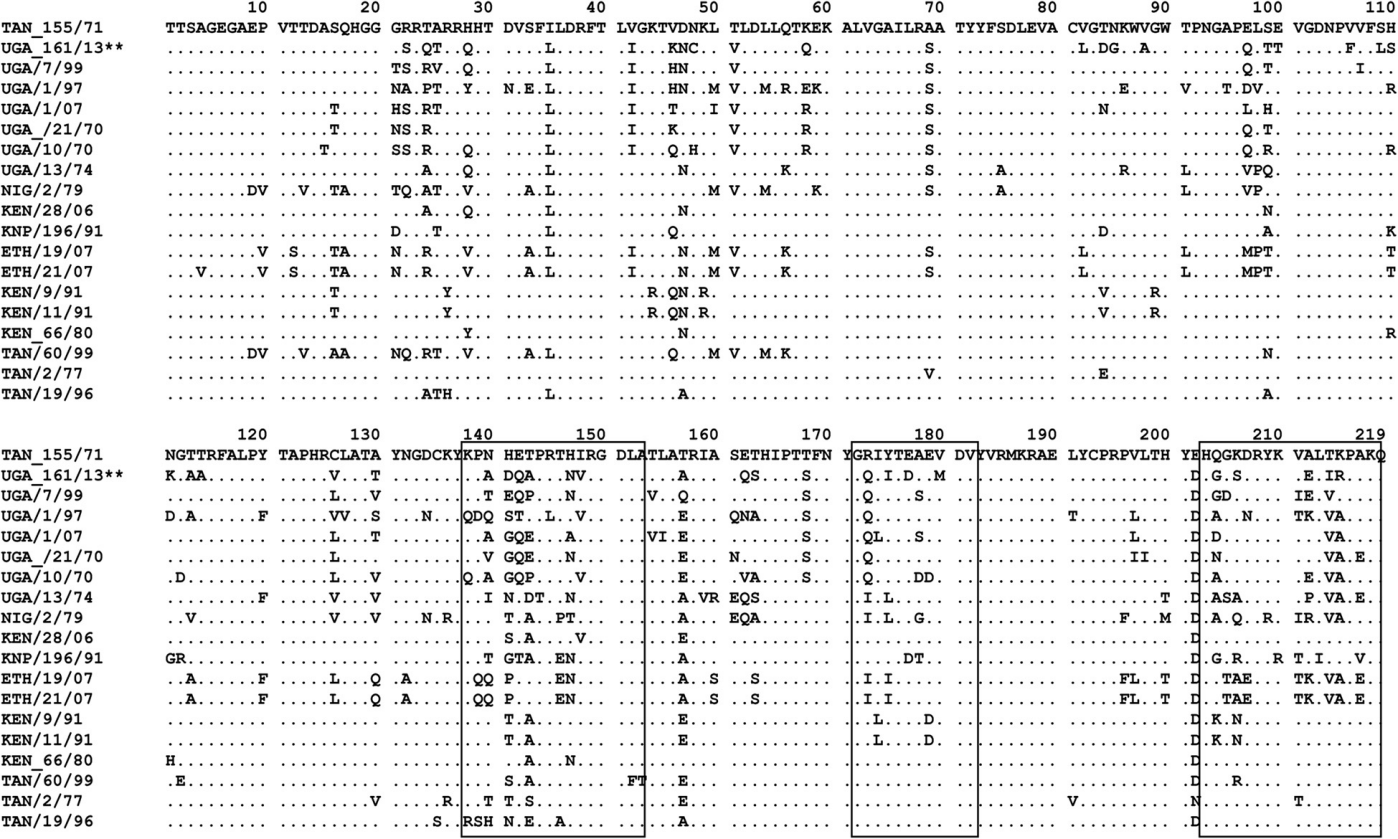
The sequence alignment for the VP1 protein of 19 SAT 1 FMD viruses. Dots indicate amino acid residues identical to the reference sequence SAT 1 TAN/155/71

Since the VP1 sequences of the two isolates from H18 were identical, the 20 aligned SAT 1 VP1 coding sequences (Table 1) encoded 19 different amino acid sequences, each with 219 amino acid residues (Fig. 3), which were conserved at 51.3 % of the sites. Multiple amino acid substitutions were observed within the known hypervariable regions [53]. In the G-H loop, compared to the vaccine strain (TAN/155/71), residues 137 and 149–151 (the receptor binding RGD motif) remained conserved in all the 19 sequences, while multiple substitutions occurred in the section including residues 138–148 which is known to be a key antigenic region in FMDVs [53]. The seven Ugandan isolates were conserved at residues 146, 152 and 153 with each having a distinct amino acid (aa) substitution at positions 140–143. UGA/116/13 showed full aa conservation with UGA/7/99, UGA/1/07, UGA/13/74 and UGA/21/70 at positions 138, 139, 144 and 145, while the UGA/1/97 and UGA 10/70 strains had different substitutions (Fig. 3). UGA/116/13 had multiple substitutions at positions 140(N → A), 141(H → D), 142(E → Q), 143(T → A), 147(H → N) and 148(I → V) compared to the vaccine strain (TAN/155/71) and distinct substitutions in these positions were also observed in the other Ugandan isolates but without a pattern. In the H-I loop (residues 172–182), all the 19 sequences were fully conserved at positions 172, 176 and 181–182. However, there were distinct substitutions among the Ugandan isolates within this region. Near the C-terminus (residues 203–219), five positions (203, 208, 216, 218 and 219) were conserved in all the 19 isolates, while the Ugandan isolates also showed full conservation at positions 209, 210 and 213 plus almost complete conservation at positions 207, 211 and 217 (Fig. 3). The UGA/116/13 differed from the other 6 strains by having substitutions outside the G-H loop (139–153), the H-I loop (172–182) and the C-terminus (203–219) (see Fig. 3).

### Herd owner replies to semi-structured questionnaires

According to the responses to questionnaires (see Additional file 1), 96 % of the herd owners grazed their herds communally inside QENP and 63 % frequently observed close contact with buffalo, especially during the dry season. Moreover, 75 % of the farmers reported that buffalo and other wildlife grazed around the cattle kraals during the night. Most farmers were aware of FMD outbreaks in their area, 58 % had experienced clinical FMD in their herds and 12.5 % had observed signs similar to clinical FMD (although not confirmed) since the last documented outbreak in 2006 (see Additional file 1). When suspected cases of FMD occurred, 63 % of the farmers reported this to the area veterinarian, 17 % drove the animals into the waters of the Albertine rift valley salt lakes, 33 % applied local herbs and 4 % treated with oxy-tetracycline sprays.

## Discussion

At the QENP livestock-wildlife interface, young unvaccinated cattle from 24 herds without reports of clinical signs of FMD had an overall seroprevalence of 15 % (37/247) for antibodies against FMDV NSPs. This is higher than the 5 % seroprevalence reported in cattle herds without clinical signs in the same area in 2006 [31] but compares well with the 12 % value reported in young calves in FMD-endemic Ethiopia [41].

Evidence for prior FMDV infection varied between herds, thus evidence for exposure to FMDV was absent in ten herds, scanty in eight herds and more substantial in six herds (25 %). The observed scattered presence of antibodies does not appear to fit well with the general appreciation of the epidemiology of FMD as an efficiently spreading disease with high morbidity in affected herds and high risk of transfer to other herds through physical contact and fomites [54]. Moreover, although the farmers were well acquainted with clinical signs of FMD, a number of them would not report outbreaks, but rather used traditional control measures like application of local herbs or walking the animals through the salt lakes known to contain common salt and sodium bicarbonate [55].

There is an undefined level of trade with cattle, including young stock for fattening, coming into the area from other districts; thus individual animals with antibodies against FMDV may be explained as animals infected elsewhere and brought into the herds as replacement stock or gifts to the owners as part of local customs [32]. However, the higher antibody prevalences in H7 and H19 and the isolation of SAT 1 FMDV from OPs from two cattle in H18 support spread within these herds (originating either from within the district or from other districts); moreover, it cannot be excluded that the infections causing antibodies in the other herds have taken place in Kasese district.

Another possibility is that some of the antibodies against FMDV observed in this study are residual maternally transferred antibodies. Since cattle in this area are likely to have their first calf at 3–4 years of age and to have several calves, it is likely that some of the dams to the sampled calf cohort (born between August 2009 and February 2011) were exposed to FMDV by infection and/or vaccination during the 2006–2007 outbreak [31, 56]. A fraction of these dams may have retained sufficient level of antibodies, especially against the NSPs, to transfer these to their offspring. Moreover, although FMD outbreaks had not been reported in the area since 2006, the District Veterinary Office noted that underreporting was suspected since quarantine restrictions due to FMD would paralyze all economic activities in the area.

Although it is not clear when, or how, some of the antibody positive animals in these herds were infected, SPBE assays showed that 11 of the 37 NSP positive samples had antibody titres ≥ 80 against one or more serotypes. Based on SPBE and VNT results from the few positive animals, it appeared that serotype O FMDV had infected some animals from H1, H14 and H19, while SAT 1 FMDV had infected some animals from H3 and possibly H18, in the latter case this was confirmed by isolation of SAT 1 FMDV from two animals. The situation was unclear in herds H19 and H23 with high SPBE antibody titres against serotypes O, SAT 1, SAT 2 and SAT 3, which could be due to the previously observed cross-reactivity between the SPBEs and also between the VNTs [31, 56]. Moreover, if animals were purchased from other districts, vaccine-derived antibodies cannot be excluded either. In accordance with previous findings in the area [31, 56], there was no conclusive serological evidence for previous infection by serotypes A, C, Asia 1 and SAT 3 in the sampled cattle, although very recently SAT 3 FMDV was isolated from an asymptomatic calf from the area [34].

The SAT 1 FMDV isolates obtained from the two healthy animals in H18 are the first reported SAT 1 isolates from Ugandan cattle since 1999 [57], while SAT 1 isolates have been obtained from buffalo in QENP in 1997 and 2007 [35, 36]. The relatively infrequent isolation of SAT 1 FMDV from outbreaks in Ugandan cattle could be the result of poor surveillance in the form of underreporting of FMD outbreaks and limited submission of samples of sufficient quality for virus isolation to the central diagnostic laboratory at the National Animal Disease Diagnostics and Epidemiology Centre (NADDEC) (Dr. Anna Rose Ademun, personal communication). Moreover, there have been limited options for typing FMDV locally in Uganda [9].

The combination of rather low C_T_ values (22 and 23) in the 5'UTR tests for the two H18 OP samples that yielded the SAT 1 FMDV isolates and SPBE antibody titres of 40 and 80 for antibodies against SAT 1 FMDV in sera from the same animals, indicate that these two animals were in the subacute stage of the infection. However, no clinical signs were observed during the sampling and there were no reports of clinical signs in this or in other cattle herds in the area. Moreover, the questionnaire responses indicated that the indigenous Ankole cattle from this area have less severe clinical signs of FMD than the pure exotic and crossbred cattle. Hence, these data support the notion that some of the Ugandan indigenous Long-horned Ankole cattle may go through FMDV infection with only minimal clinical signs of FMD. This is consistent with the very recent isolation of SAT 3 FMDV from an OP sample from a clinically healthy young calf in the QENP area [34] and has been previously reported for indigenous cattle in Botswana [58, 59]. If true, this notion has a bearing on the passive surveillance reporting system for FMD in Uganda that is based on clinical observations.

This study shows a considerable genetic difference between the current Ugandan SAT 1 cattle virus and the vaccine strain (TAN/155/71) that is currently used to control FMD outbreaks caused by SAT 1 FMDV in Uganda, indeed these belong to different topotypes. Thus, vaccine matching studies using the currently circulating SAT 1 strain are required. In addition, the observed differences from earlier buffalo and cattle isolates imply that different SAT 1 lineages have been in circulation in this area over the last 50 years. To verify this, more Ugandan FMDV SAT 1 isolates should be collected and characterised.

Despite the close interaction that occurs between cattle and buffalo at the QENP livestock-wildlife-interface, this study did not provide evidence to support the view that the FMD viruses circulating in cattle in this area originate from wildlife as has been observed in South Africa [60]. This could be attributed to the limited numbers of isolates from cattle and the long time-intervals between their isolations; but it also does not exclude the possibility that separate pools of viruses circulate in cattle and wildlife. The VP1 coding sequences of five of the six Ugandan SAT 1 isolates were different from those of isolates from other countries, indicating that the Ugandan SAT 1 strains may be geographically limited to Uganda as previously suggested [61].

## Conclusions

Although FMD was last reported in Kasese District in 2006, evidence of infection with FMDV was found in six of 24 cattle herds from the QENP livestock-wildlife-interface. The SAT 1 FMDV isolated from one of these six herds was markedly different from the earlier buffalo isolates from the same area; thus transmission between buffalo and cattle could not be proven. The genetic difference (ca. 26 % within the VP1 coding region) between the current SAT 1 isolates and the vaccine strain used to control FMD outbreaks in the country calls for vaccine matching to ensure the effectiveness of vaccination in this region. Additional surveillance and characterization of field strains from cattle and buffalo at this livestock-wildlife interface is necessary for monitoring of emerging strains and to enhance the understanding of the role of wildlife in the epidemiology of FMD in cattle in East Africa.

## Additional file

Additional file 1:
**The questionnaire used in the survey.** (DOCX 14 kb)

## Abbreviations

Amplitaq Gold: Thermostable DNA polymerase enzyme from thermophilic bacterium *Thermusaquaticus*
BLAST: Basic Local Alignment Search Tool
BOT: Botswana
bp: Base Pair
BTY: Bovine thyroid cells
cDNA: Complementary Deoxyribonucleic acid
CPE: Cytopathic effect
Ct: Cycle threshold
DNA bases used: Adenine (A), Guanine (G), Cytosine (C) and Thymine (T)
DNA: Deoxyribonucleic acid
dNTP: Deoxyribonucleotide triphosphate
EA: East Africa
ELISA: Enzyme-Linked Immunosorbent Assay
FMD: Foot-and-mouth disease
FMDV: Foot-and-mouth disease Virus
G-H loop: A highly variable region within VP1 of FMDV (ca. 140–160) but which includes the highly conserved RGD motif required for receptor binding
H: Herd
MA: Massachusetts
MEGA: Molecular Evolutionary Genetic Analysis
MgCl2: Magnesium Chloride ions
MUSCLE: Multiple sequence alignment method for amino acids and nucleotides
NADDEC: National Animal Disease Diagnostics and Epidemiology Centre
NJ: Neighbor joining
NSP: Non-Structural Proteins
OD: Optical Density
ODP: Optical density percent
OP: Oro-pharyngeal fluid
PBS: Phosphate buffered saline
PCR: Polymerase chain reaction
QENP: Queen Elizabeth National Park
RLT buffer: A lysis buffer for lysing cells and tissues before RNA separation
RNA: Ribonucleic acid
RT-qPCR: Reverse transcription quantitative polymerase chain reaction
SAT: Southern African Territories
SPBE: Solid phase blocking ELISA
TAN: Tanzania
UGA: Uganda
USA: United States of America
UTR: Untranslated region
VNT: Virus neutralization test
VP: Virus protein
ZIM: Zimbabwe
3D protein: RNA dependent RNA polymerase of FMDV
