# Supplementary material for: Unrecognized circulation of SAT 1 foot-and-mouth disease virus in cattle herds around Queen Elizabeth National Park in Uganda
Source: BMC Vet Res. 2016 Jan 6;12:5. doi: 10.1186/s12917-015-0616-1 (PMC4704403; doi:10.1186/s12917-015-0616-1)
Supplement: Additional file 1: — The questionnaire used in the survey. (DOCX 14 kb) [file 12917_2015_616_MOESM1_ESM.docx]

**Additional file 1: Animal/herd owner FMDV questionnaires**

**Livestock-Wildlife interface FMD survey questionnaire at Queen Elizabeth National Park**.

This questionnaire is aimed at collecting data from the cattle owners who will consent to participate in this study on the epidemiology of FMDV at the wildlife interface. The results of this study wwill be used to inform decision making as far as FMD control is concerned. The results of this study from your responces will not be disclosed to anyone, but they can be communicated to you upon request. We shall be gratefull if you allow us to carry out this survey on your herd.

Respondent questionnaire Number….... Date of interview........................ District ...................Subcounty...............................Parish/village…...............................

Owners/respondents Name........................................................................................................

Herd number............................. GPS position. ........................................................................

1. How many animals do you have on the farm/in your herd?
2. How do youkeep your animals/ the farming method practiced: Communal grazing-1; Intensive grazing-2; Zero grazing- 3
3. What is the type of animals kept: Local Ankole......1; Cross breeds.....2; Buffaloes…...3. others (specify) 4…………….
4. Have you ever heard of FMD? Yes.....1. No..... 2.
5. If yes, has it ever occurred in your parish? Yes.....1. No.........2.
6. When did this occur? Options (Before 2007........1 After 2007..........2)
7. If yes, where exactly did it occur: On your farm...1: Within your village...2: In neighbouring villages within our parish…......3.Far away from here……...4
8. Which animals were affected: Domestic: 1 (Cattle, goats, sheep):
9. Wildlife. 2. (Buffaloes, antelopes, others)
10. What age group of domestic animals was most affected? Adults (3-4yrs).1 Yearling (1-2yrs).2. Calves (< 12months).3.
11. How do you think the disease got into your herd/ village/parish? Purchased animals..... 1; via contact with wildlife......... 2; From Congo..... . 3. Not aware ............ 4.
12. How do you think FMD is a problem to you? ......................................................................
13. What other diseases do you consider as being important in your animals?
14. Listed as per the herdsman information (TBD, Brucellosis, Trypanosomiasis, Anaplasmosis, ECF any others etc). 1........................... 2...............................3............. 4............................6..................
15. Rank them from the most feared to the least. (Participatory)
16. How often do your animals have contact with wildlife in the National Park?

Daily ..........1: Regularly............... 2: Never at all............. 3.

1. If they do, which wild animals do they mix with (specify)?.............
2. Which period of the year does this contact occur ...........................................
3. Does this contact cause any problems to your flocks? Yes.......1 None..............2.
4. If yes, what are those problems......................
5. Do you think wildlife has a role in the spread of this FMD in your Cattle? ....... If yes, explain..............
6. What actions are taken when you suspect its FMD? (The mobile farmers/herdsmen ....................................
7. Do you report? Yes .......1 No.........2.
8. To whom do you report and where? To the Local Council .................1 : To the Subcounty HQ...........2 ;To the area Vet......3; To the commissioner direct.......................4
9. If you have ever had FMD how did you treat your affected animals?
10. Local rock salt...1; Injections... 2; Sprays /aerosols... 3; Called a vet to help ...4; others specify ..............
11. Are your animals vaccinated against FMD? Yes......1 No......2
12. How many times have your animals been vaccinated against FMD? .................................
13. When they were last vaccinated? ........................................................................................
14. Who did the vaccination? Govt vet.....1; Community Animal Health Worker...........2; Fellow herdsmen ................3
15. Did you see any signs of FMD after this vaccination? ...................................................
16. Do you ever buy new cattle to add onto your flock? Yes......1 No..........2......
17. If yes, how often...................................................................................................................
18. Have you ever moved your animals to Congo and back? Yes.......1.No........2.
19. What prompted you?
20. Do you practice fattening yearlings for sale?

Thank you.
